# Identification and Characterization of Novel Perivascular Adventitial Cells in the Whole Mount Mesenteric Branch Artery Using Immunofluorescent Staining and Scanning Confocal Microscopy Imaging

**DOI:** 10.1155/2012/172746

**Published:** 2012-02-19

**Authors:** Chandra Somasundaram, Rahul K. Nath, Richard D. Bukoski, Debra I. Diz

**Affiliations:** ^1^Cardiovascular Disease Research Program, JLC-Biomedical/Biotechnology Research Institute, North Carolina Central University, Durham, NC 27707, USA; ^2^Research Division, Texas Nerve and Paralysis Institute, Houston, TX 77030, USA; ^3^Intron Pharmaceuticals, Houston, TX 77005, USA; ^4^Hypertension & Vascular Research Center, Wake Forest University School of Medicine, Winston-Salem, NC 27157, USA

## Abstract

A novel perivascular adventitial cell termed, adventitial neuronal somata (ANNIES) expressing the neural cell adhesion molecule (NCAM) and the vasodilator neuropeptide, calcitonin gene-related peptide (CGRP), exists in the adult rat mesenteric branch artery (MBA) in situ. In addition, we have previously shown that ANNIES coexpress CGRP and NCAM. We now show that ANNIES express the neurite growth marker, growth associated protein-43(Gap-43), palladin, and the calcium sensing receptor (CaSR), that senses changes in extracellular Ca(2+) and participates in vasodilator mechanisms. Thus, a previously characterized vasodilator, calcium sensing autocrine/paracrine system, exists in the perivascular adventitia associated with neural-vascular interface. Images of the whole mount MBA segments were analyzed under scanning confocal microscopy. Confocal analysis showed that the Gap-43, CaSR, and palladin were present in ANNIES about 37 ± 4%, 94 ± 6%, and 80 ± 10% respectively, comparable to CGRP (100%). Immunoblots from MBA confirmed the presence of Gap-43 (48 kD), NCAM (120 and 140 kD), and palladin (90–92 and 140 kD). In summary, CGRP, and NCAM-containing neural cells in the perivascular adventitia also express palladin and CaSR, and coexpress Gap-43 which may participate in response to stress/injury and vasodilator mechanisms as part of a perivascular sensory neural network.

## 1. Introduction

Vascular growth and remodeling occur in association with certain physiological and pathological conditions. In addition, vascular regeneration and repair are essential for the survival of blood vessels. These processes involve numerous cell types. There are still uncharacterized and less characterized cell types in vascular adventitia, which include vascular stem/progenitor cells [1–10] and adventitial neuronal somata (ANNIES) [11]. The vascular adventitia is a complicated tissue [12], which is found to be the most active layer in terms of cell turnover [13]. In addition, within the vascular adventitia resides amyelinated nerves known as “nerva vasorum” [14]. Using fluorescence confocal microscopy (FCM) to visualize vascular wall 3D organization of different cellular and extracellular elements of the intact artery with minimal 3D distortion [11, 13], we recently demonstrated ANNIES coexpressing neural cell adhesion molecule (NCAM) and calcitonin gene-related peptide (CGRP) in the adult rat mesenteric branch artery (MBA). These cells can be enzymatically dispersed and maintained in culture [11].

The present study was designed to further characterize ANNIES in adult rat MBA as cells with axonal and neurite growth markers, such as growth-associated protein-43 (Gap-43) and palladin, and to determine whether they express the calcium sensing receptor (CaSR). Recently, CaSR mRNA and protein expression has been demonstrated in rat whole MBA and other vascular tissue extracts, also shown in vascular smooth muscle cells in culutre [15, 16]. None of these studies show the specific cell(s) in the artery *in situ *expressing the CaSR. We demonstrate here that nucleated ANNIES cells together with nerve processes clearly express palladin, CaSR, and coexpress Gap-43 protein in the whole mount MBA by immunofluorescent staining followed by laser confocal analysis. In addition, we confirm the expression of these markers using Western blot analysis.

## 2. Materials and Methods

### 2.1. Animals

All procedures using laboratory animals were reviewed and approved by the Institutional Animal Care and Use Committee of North Carolina Central University. Male Wistar rats, 10–12 weeks of age, were obtained from Harlan Sprague Dawley (Indianapolis, USA). All animals were continually monitored, and upon arrival, they were maintained in colony rooms with fixed light: dark cycles and constant temperature and humidity and provided with Purina rodent chow (Harlan Teklad, Madison, Wis, USA) and water ad libitum.

### 2.2. Preparation and Isolation of Vessels

Mesenteric arteries were dissected from rats (*n* = 10) as previously described [11]. In brief, rats were deeply anesthetized with 2% isoflurane and then sacrificed by open chest cardiac puncture. The small intestine and all vessels feeding it were removed in block and placed in cold physiological salt solution (PSS). Branch I and II arteries were carefully dissected from the surrounding fat and mesenterium, taking care to leave a portion of the omental membrane attached to the vessel. A 12 *μ*m-diameter stainless steel wire was inserted into the lumen to remove blood and to serve as a handle for moving the vessel segment between solutions.

### 2.3. Immunostaining and Confocal Analysis

Vessels of MBA were fixed in buffered formalin for 20 min and washed three times in TBS (Tris-buffered saline). The vessels were kept in blocking solution containing 8% BSA in TBS and incubated with primary antibody such as polyclonal anti-Gap-43, anti-CaSR (Molecular probes), anti-palladin (gift from Dr. Otey, UNC-Chapel Hill) overnight at 4°C. For dual immunofluorescence staining anti-CGRP (Phoenix Pharmaceuticals, Calif), and anti-Gap-43 (Molecular probes) were incubated for 2 h at room temperature (RT). After incubation, the vessels were washed thrice in TBS and incubated with appropriate secondary antibody tagged with alexa fluor 647 alone, and with 488 in case of co-staining for 1 h at RT. After washing thrice in TBS, vessels were incubated with the nuclear stain Sytox (Petticoat Junction, OR) to identify the nuclei of ANNIES. Vessels were mounted on glass slides in a glycerol-based Antifade medium (Molecular probes) after washing. Segments were viewed with a Zeiss LSM 510 confocal microscope (Zeiss Instruments) with 100x, 40x, and 20x oil immersion objectives.

We considered the number of CGRP staining cells as 100% of ANNIES, and calculated the percentage of ANNIES expressing the other markers (Gap-43, CaSR and palladin) in comparison to the CGRP expressing cells.

### 2.4. Western Blotting

Protein was extracted from minced MBA (*n* = 5) by homogenizing in a ground-glass homogenizer (MBA) in buffer containing 10 mM Tris, pH 7.5, 0.25 M sucrose, 3 mM MgCl_2_ containing 1% (v/v) Triton X-100, dithiothreitol (1 mM), Pe-fabloc (1 mM), leupeptin (10 *μ*M), bestatin (130 *μ*M), pepstatin (1 *μ*M), and calpain inhibitor II (10 *μ*g/mL). The homogenate was then centrifuged at 16,000 g for 10 min and the pellet was used for NCAM, Gap-43 and palladin. The pellet was dissolved in buffer containing 10 mM Tris, pH 7.5, and 1% Triton X-100, size separated using 8% SDS-PAGE, and electroblotted onto nitrocellulose membrane (Bio-Rad Laboratories, CA) as described [17]. The membrane was then probed with antibodies separately (Chemicon International Inc., Calif) and visualized using an HRP-conjugated secondary antibody, and the chemiluminescence method (Amersham Pharmacia Biotech, NJ).

## 3. Results and Discussion

### 3.1. Immunofluorescent Analysis of the Whole Mount MBA by Confocal Microscopy

**Figure 1:**
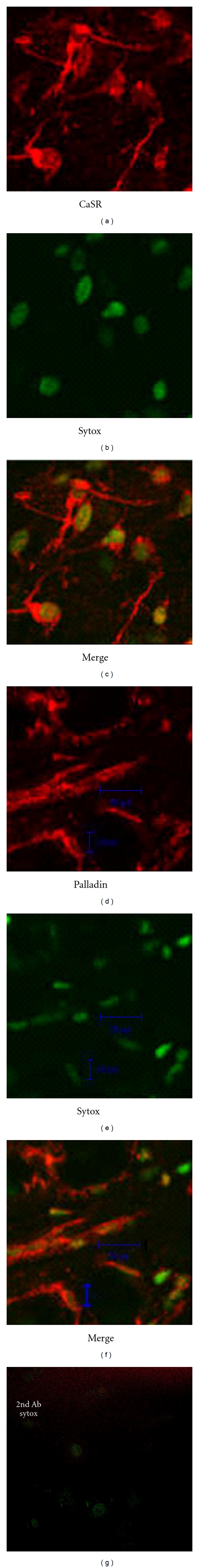
Immunofluorescent confocal analysis of the whole mount rat MBA showing the CaSR and palladin expression. ANNIES and the nerve fibers strongly express (94 ± 6%) the CaSR protein (top panel—in red, alexa fluor—647). Fibroblast like cells with no processes, and cells with nerve fibers (ANNIES) showing palladin expression (80 ± 10%) in red (lower panel). Nuclei stained in green (sytox)—(100x), (g). Rat MBA stained for secondary antibody alone, tagged with alexa fluor-647, and nuclear stain sytox—(100x). (Representative of 3 experiments.)

We used the whole mounts of the adult rat MBA and immunofluorescence confocal microscopy imaging to further characterize ANNIES in arterial adventitia. The use of whole-mount MBA from rats enabled us to have an accurate reconstruction of the cellular and nerve interrelationships and innervation patterns of perivascular adventia and vasa nervorum within the MBA. This also facilitates the ability to study the expression pattern of the neurochemical markers in situ, without the need to cut them as with conventional histology. Furthermore, this approach allows us to localize and visualize the morphology/phenotype and quantify these cells (ANNIES) and nerve structures *in situ *coexpressing vasodilator trophic factor CGRP and NCAM [11], and axonal growth markers Gap-43 (Figure 3) as well as CaSR (Figure 1). CaSR is shown to play significant role in blood pressure regulation by releasing an unknown vasodilator [18]. More recently, Weston et al. [19] reported that this vasodilator is still unidentified.

### 3.2. ANNIES and Perivascular Nerve Fibers in Rat MBA Express CaSR

We hypothesize that the vasodilator released by CaSR, which is still unidentified [19], might be the potent vasodilator neuropeptide, CGRP. Our present finding demonstrates that 94 ± 6% (*n* = 3) ANNIES cell bodies express the CaSR (Figure 1), compared with the ANNIES identified by the expression of CGRP as previously demonstrated [11]. The CaSR is shown to regulate the production of CGRP [20] that is involved in the regulation of the release of the neurotransmitter in the synaptic space [20]. Our laboratory has demonstrated that the CaSR mediates Ca2+ induced relaxation of isolated mesenteric arteries [18] via an unknown vasodilator substance, which is independent of endothelium and NO [18]. These previous findings support our hypothesis that this putative vasodilator released by the CaSR might be the CGRP expressed in ANNIES and perivascular nerve fibers [11]. We demonstrate here that nucleated ANNIES cells and the nerve processes strongly express the CaSR.

### 3.3. Rat MBA and ANNIES Express Gap-43, Palladin and Coexpress CGRP

Gap-43 is a marker of neural outgrowth and regeneration [21]. It is shown to locate in the growth cones of growing neurites, where it interacts with F-actin associated adhesion molecule and/or extracellular matrix complexes to promote neurite extension [22]. In addition, it has been shown that the phosphorylated Gap-43 stabilizes long actin filaments and has the ability to directly influence the structure of the actin cytoskeleton response to extracellular signals [23]. Similarly, palladin has been shown to express in the growth cone and colocalize with focal adhesion [24] and respond to vascular [25] and dermal injury [26].

**Figure 2:**
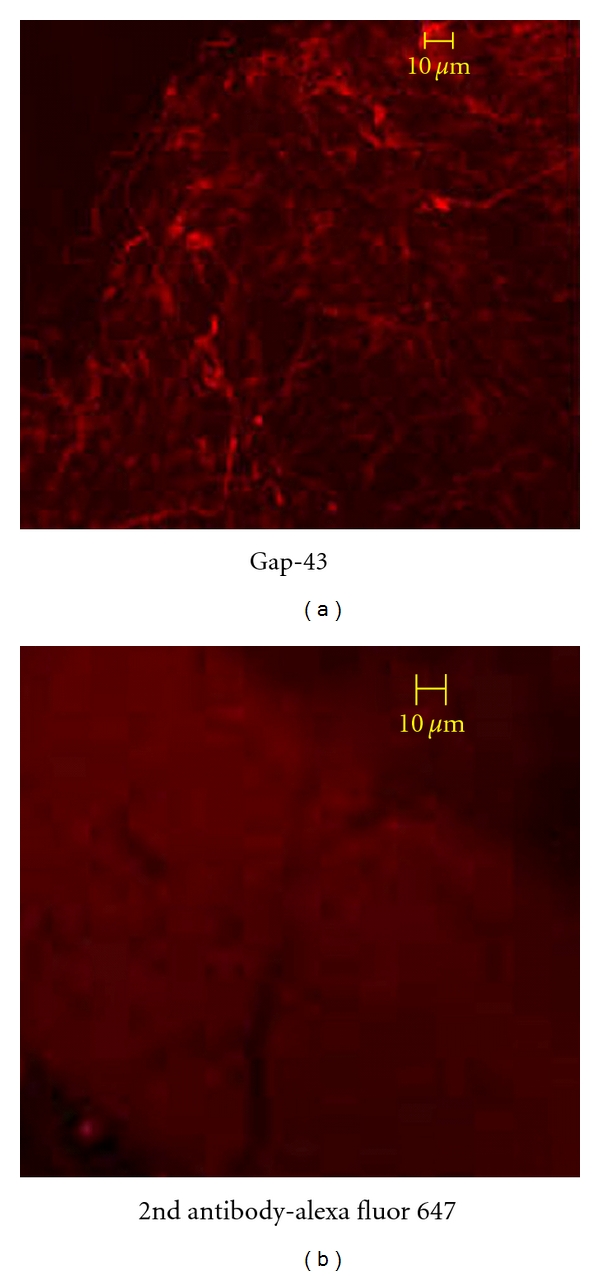
Immunofluorescent confocal analysis of the whole mount rat MBA expressing Gap-43. The whole mount of adult rat mesenteric arterial adventitia expresses 37 ± 4% Gap-43 (in red, alexa fluor-647) under scanning fluorescent confocal microscopy—20x. (Representative of 3 experiments.)

**Figure 3:**
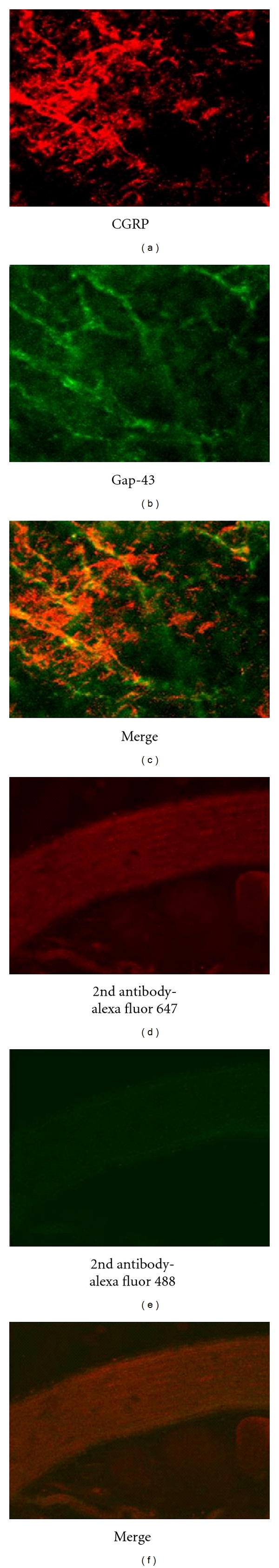
Immunofluorescent confocal analysis of the whole mount rat MBA expressing CGRP (in red, alexa fluor-647), Gap-43 (in green, alexa fluor-488), and the coexpression of these two markers (yellow). ANNIES and the nerve fibers strongly express CGRP (100%), and Gap-43. (Representative of 3 experiments.)

Confocal analysis of the whole mount MBA in our experiment showed that Gap-43 is strongly distributed in 37 ± 4% of the perivascular nerve fibers (Figure 2, *n* = 3), and coexpressing CGRP (Figure 3), also identified by the expression of CaSR (Figure 1). Palladin is present in both SMC [25] and ANNIES in the rat MBA (Figure 1). Palladin expression is apparent in a mixed population of fibroblasts and in cells with nerve processes, ANNIES (Figure 1) that were shown to coexpress CGRP and NCAM [11]. The CaSR and palladin were present in ANNIES about 94 ± 6% and 80 ± 10% (*n* = 3), respectively. Strong immunofluorescence for NCAM [11], and axonal growth markers such as Gap-43 as reported in this paper, revealed the presence of many neuronal cell bodies within the vasa nervorum of rat MBA. In addition, the presence of Gap-43 in ANNIES indicates that ANNIES shows features of sensory neurons of the DRG, undergoing responses to stress/injury, given that the expression of Gap-43 in DRG cells is increased in response to injury [27, 28]. In addition, CGRP induces schwann cell proliferation, and thus thought to be involved in peripheral nerve injury and repair [29].

### 3.4. Western Blot Analysis of MBA Expressing NCAM, Gap-43, and Palladin

**Figure 4:**
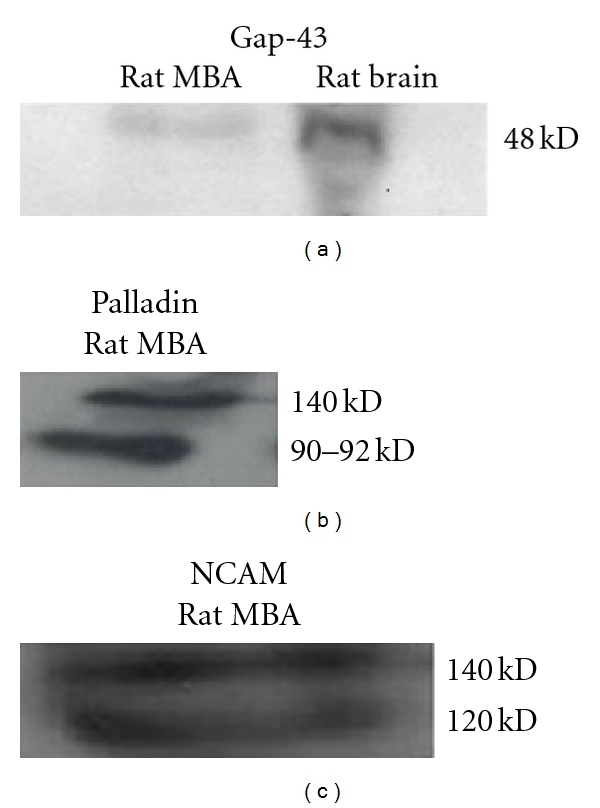
Western blot analysis of Gap-43, palladin, NCAM in adult rat MBA. Immunoblots from the rat MBA protein lysate also confirmed the presence of Gap-43 palladin and NCAM. Palladin expresses as two isoforms of 90–92 and 140 kD proteins. Gap-43 antibody detected a ~48 kDa protein, whereas anti-NCAM detected 120 and 140 kD proteins. (Representative of 3 experiments.)

Immunoblots from the rat MBA also confirmed the presence of Gap-43 (Figure 4) and palladin as two isoforms of 90–92 and 140 kD (*n* = 3). This protein pattern is different from other adult tissues such as brain that expresses three isoforms (90–92, 140, and 200 kD) or SMC where only one isoform (90–92 kDa) is expressed [30]. CaSR protein expression by western blot in the protein extract of the whole mesenteric artery was reported [31]. We previously demonstrated beta-CGRP expression in rat MBA by RT-PCR, cloning, and sequence analysis [11].

Cells that are not with nerve fibers, probably fibroblasts, were also stained for palladin as it has been reported by other investigators [30]. There is no nerve like structures around some of the fibroblast like cells that express palladin. In addition, NCAM, CGRP [11], and Gap-43 are expressed in ANNIES, but these markers are not shown to express in the other adventitial fibroblasts, smooth muscle, and endothelial cells.

## 4. Conclusion

This is the first report that cells in the peripheral vasculature with a neuronal phenotype express markers of active neurite growth. The presence of CGRP-containing neural cells in the vascular adventitia may participate in response to injury and vasodilator mechanisms as part of a perivascular sensory neural network. ANNIES cell morphology with nerve fibers and expression of neural and neurite growth markers reveal that these cells are distinct from the other known cells in blood vessel. The additional finding that the CaSR is associated with ANNIES suggests that these cells may participate in the regulation of myogenic tone.
